# Prevalence of Intestinal Parasitic Infections Among Students Attending Schools in La'elay Maichew District, Tigray Region, Ethiopia

**DOI:** 10.1002/hsr2.72609

**Published:** 2026-06-07

**Authors:** Teklay Abrha Yanshet, Gebremedhin Gebrezgabiher, Melese Birmeka

**Affiliations:** ^1^ Department of Biology, College of Natural and Computational Sciences Hawassa University Hawassa Sidama Ethiopia; ^2^ Department of Biology, College of Natural and Computational Sciences Samara University Samara Afar Ethiopia; ^3^ Department of Veterinary Medicine, College of Veterinary Medicine and Animal Sciences Samara University Samara Afar Ethiopia

**Keywords:** Ethiopia, intestinal parasites, La'elay Maichew, prevalence, risk factors, school students

## Abstract

**Background and Aims:**

Intestinal parasitic infections (IPIs) remain a significant public health challenge across Ethiopia, including the Tigray region, where school‐age children (SAC) are particularly vulnerable. The La'elay Maichew district, characterized by rural agrarian and irrigated settings and the 2020–2022 armed conflict, may experience heightened transmission of IPIs. This study aimed to assess the status of IPIs and associated risk factors among school students in La'elay Maichew district, Tigray Region, Ethiopia.

**Methods:**

A school‐based cross‐sectional study was conducted in June 2024, involving 190 school students aged 7–21 years. Data were collected via structured questionnaires, and stool samples were processed using the Kato–Katz technique.

**Results:**

This study found an overall prevalence of IPIs of 3.2% (6/190) among school students [95% CI: 1.2–6.9], with *Enterobius vermicularis* (1.6%), *Ascaris lumbricoides* (1.1%), and *Taenia saginata* (0.5%) identified as the primary species. Those children aged 15–21 years and grades 9–12 showed higher infection rates (7.6% and 9.3%, respectively), though multivariate analysis revealed no significant associations. Behavioral risk factors, including poor handwashing (76.3% before/after meals, 82.1% after toilet use), lack of toilet use (75.8%), and barefoot walking (80.5%), were prevalent but not statistically linked to infection status.

**Conclusion:**

The low prevalence observed in this study may reflect the sustained impact of previous mass drug administration (MDA) programs in the study area. It could also result from post‐conflict population displacement, changes in school attendance patterns, or methodological limitations, particularly the use of a single Kato–Katz thick smear, which has lower sensitivity for detecting light‐intensity infections. Persistent poor hygiene practices highlight the need for integrated interventions that combine deworming with improved water, sanitation, and hygiene (WASH) education to sustain low infection rates and prevent resurgence in the study area.

## 1 Introduction

Intestinal parasitic infections (IPIs), caused by a range of helminths and protozoa, remain among the most widespread infections affecting humans [1, 2]. Globally, an estimated 1.5 billion people are affected with IPIs [3], including *Ascaris lumbricoides*, *Trichuris trichiura*, and hookworms, with the highest burden concentrated in tropical and subtropical areas where environmental, socioeconomic, and infrastructural conditions favor sustained transmission [4, 5]. Over the past two decades, global control efforts, particularly those targeting soil‐transmitted helminths (STHs), have reduced infection prevalence and intensity in several endemic countries. Despite progress, reductions have been uneven, and many endemic countries, particularly in Sub‐Saharan Africa, continue to report persistent or recrudescent transmission in vulnerable populations [6].

In Sub‐Saharan Africa, and specifically in Ethiopia, the epidemiology of IPIs over the last two decades reflects both progress and persistent challenges. Prior to large‐scale preventive chemotherapy programs, prevalence of STH infections in Ethiopian school‐age children (SAC) frequently exceeded 50% in many settings [7]. Following the scale‐up of national neglected tropical disease (NTD) control programs, including mass drug administration (MDA) initiated around 2013 [8], several studies have documented reductions in infection prevalence and intensity [9]. Nevertheless, more recent systematic reviews and meta‐analyses indicate that IPIs remain highly prevalent, with pooled estimates among Ethiopian children still ranging between 30% and 50%, and considerable heterogeneity across regions [10, 11]. This suggests that although MDA has reduced morbidity, transmission persists in settings where reinfection occurs rapidly.

A growing body of evidence highlights that sustained reductions in IPIs require not only chemotherapy but also improvements in environmental and behavioral determinants. Studies across Ethiopia and similar low‐ and middle‐income countries (LMICs) consistently identify inadequate sanitation, unsafe water sources, poor hygiene practices, and low socioeconomic status as key drivers of persistent transmission [12, 13]. In particular, lack of access to improved latrines, open defecation, irregular handwashing practices, and exposure to contaminated soil and water have been strongly associated with higher infection risk among children [10, 11]. Importantly, while MDA programs have expanded over the past decade, evidence suggests that their effectiveness is often undermined by high reinfection rates in communities where water, sanitation, and hygiene (WASH) conditions remain inadequate [12]. Recent studies indicate that the effectiveness of MDA may decline over time without complementary WASH interventions, highlighting the need for integrated control strategies [13].

School children remain the primary target group for IPI control programs due to their disproportionately high burden of infection and their central role in transmission dynamics. Biologically, children have not yet developed partial immunity to many parasitic infections, while behaviorally they are more likely to engage in activities that increase exposure, such as playing in contaminated environments, walking barefoot, and practicing inadequate hygiene [14]. IPIs in children can lead to substantial consequences, including malnutrition, impaired physical growth, reduced cognitive development, and poor academic performance [15, 16, 17], underscoring the need for targeted control and monitoring strategies.

In Ethiopia, despite over a decade of MDA implementation [18], marked spatial heterogeneity in IPI prevalence persists [10]. Variations in environmental conditions, sanitation infrastructure, access to safe water, and health service delivery contribute to substantial differences in infection patterns across regions. The Tigray region exemplifies this heterogeneity, with previous studies reporting prevalence estimates among SAC ranging widely from below 10% to over 60% depending on locality [19, 20]. This variability highlights the need for localized epidemiological data to guide targeted interventions.

More recently, the epidemiological landscape in Tigray has been profoundly influenced by armed conflict that began in November 2020. The conflict severely disrupted healthcare systems, including routine deworming campaigns, which halted for nearly 3 years [21]. Concurrently, damage to water supply systems, sanitation infrastructure, and school‐based health programs has likely increased exposure to infection risks. Evidence from other humanitarian and post‐conflict settings suggests that such disruptions can lead to resurgence or increased transmission of parasitic infections due to breakdowns in preventive and control measures [22]. However, the effect of these disruptions on the current disease burden remains uncertain. Although these conditions may increase transmission, it is also plausible that the sustained impact of pre‐conflict MDA and earlier improvements in WASH may have contributed to a residual reduction in infection burden. Nevertheless, empirical data on the post‐conflict burden of IPIs in Tigray remain limited, creating uncertainty about the current epidemiological situation of IPIs in the region and underscoring the need for updated evidence.

At the local level, environmental and livelihood factors may further influence transmission dynamics. The Dura *kebele* in La'elay Maichew district, located in Tigray region, is characterized by a predominantly rural agrarian economy, unimodal rainfall patterns, reliance on subsistence farming, and increased use of irrigation systems supported by the Mai‐Nigus dam [23]. While irrigation enhances agricultural productivity, it may also create favorable ecological conditions for parasite transmission by increasing contact with contaminated soil and water. In the post‐conflict context, intensified farming and irrigation activities as a coping mechanism for food insecurity may further elevate exposure risks among children, particularly in settings where sanitation infrastructure remains compromised.

Although numerous studies in Ethiopia and other LMICs have identified behavioral and environmental determinants of IPIs, including poor hand hygiene [16], untrimmed or dirty fingernails [16, 24], lack of hand washing after toilet use [16, 25], hand washing practice before meals or food handling [16], open defecation practices [16], absence of household latrine [26], consumption of raw or improperly washed vegetables [26], and barefoot walking [16, 26], there is a critical lack of recent, context‐specific evidence from post‐conflict settings such as La'elay Maichew district. Existing studies in the region largely focus on the interruption of healthcare services, MDA programs, and the widespread disruption of WASH services [21, 27, 28, 29], limiting their relevance to current public health planning. Therefore, there is a clear need for updated, localized epidemiological data to understand the current prevalence and determinants of IPIs in this setting. This study aims to determine the prevalence of IPIs among school students in La'elay Maichew district, Tigray region, Ethiopia, and to identify key behavioral and environmental factors associated with infection in the post‐conflict context. This study aims to provide evidence to inform targeted interventions and support the design of integrated parasite control strategies during public health recovery efforts. We hypothesized that the prevalence of IPIs would be higher among older children, those with poor hygiene practices (e.g., inadequate handwashing and untrimmed fingernails), children who walk barefoot, and those living in households lacking access to improved sanitation facilities.

## 2 Methods

### 2.1 Study Area and Selection

**Figure 1:**
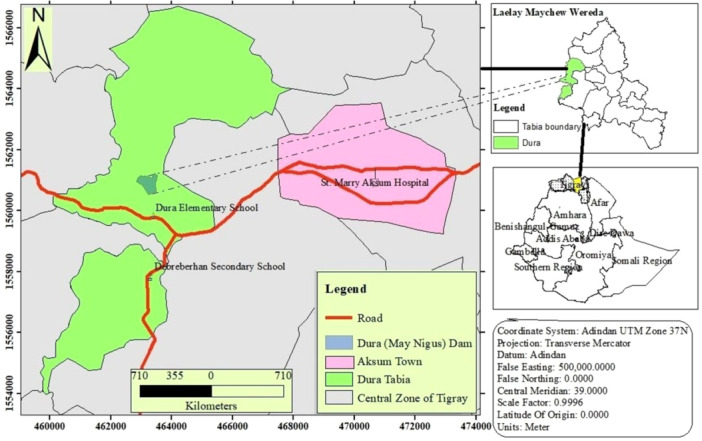
Location map of the study area.

The study was conducted in Dura Tabiya (*Kebele*) of La'elay Maichew district, located in the Tigray region, Ethiopia (Figure 1). The study *kebele* is situated 7 km west of Axum town at an elevation of 2131 m above sea level. The area experiences a unimodal rainfall pattern, with the main rainy season occurring from June to August and an average annual rainfall of approximately 662.7 mm. Dura *Kebele* hosts the Mai‐Nigus dam, a long‐standing man‐made irrigational dam, constructed in 1998 by the Commission for Sustainable Agriculture and Environmental Rehabilitation in Tigray. The dam supports local agricultural production and irrigates approximately 310 hectares of farmland. These irrigation activities, together with subsistence farming practices, may increase exposure to contaminated soil and water, thereby influencing IPI transmission. Two public schools located within the *kebele―*Dura Elementary School and Debrebrhan Secondary School―served as the study site. These schools were purposively selected because they are the only public primary and secondary schools serving the Dura *kebele* and therefore collectively represent the majority of school‐attending children in the area. A detailed description of the study area is found elsewhere [30].

### 2.2 Study Design and Period

A school‐based cross‐sectional study was conducted in June 2024 to assess the prevalence of IPIs and associated risk factors among students attending the two schools in Dura *kebele* of La'elay Maichew district, northern Ethiopia.

### 2.3 Study Population

The study population comprised students aged 7–21 years attending primary school (Grades 1–8) and secondary school (Grades 9–12). Although the typical school‐age range is 5–14 years, students up to 21 years of age were included because delayed school enrollment and grade repetition are common in rural Ethiopian settings, and particularly due to interrupted schooling and conflict‐related displacement in the study setting. For clarity, the study population is therefore referred to as school students rather than strictly SAC. School‐based surveys are commonly used as proxy indicators of community‐level IPI transmission.

### 2.4 Inclusion and Exclusion Criteria

Inclusion criteria: Students were eligible for participation if they were aged between 7 and 21 years, enrolled in one of the two selected schools, provided verbal consent, and whose parents or legal guardians provided written informed consent.

Exclusion criteria: Students were excluded if they declined participation, did not provide stool samples, or whose parents or guardians declined consent.

### 2.5 Sample Size Determination and Sampling Procedure

The sample size was determined using the single population proportion formula [31] as described in our previous work [30]:

$$n=\frac{z^{2}p(1-p)}{d^{2}}$$

where

- “*n*” represents the required total sample size,
- “*z*” is the standard normal value at a given confidence level,
- “*p*” is the estimated prevalence of *S. mansoni* infection, and
- “*d*” is the margin of error.

As this study aimed to assess both the epidemiology of *Schistosoma mansoni* and intestinal parasitic infections, an expected prevalence (*p*) of 14%, derived from a previous study on *S. mansoni* [32], was used for the sample size calculation, assuming that the prevalence of IPIs were expected at similar prevalence levels. With a 95% confidence level (*Z* = 1.96) and a 5% margin of error (*d* = 0.05), the calculated sample size was 185. After adding a 10% non‐response rate, the final sample size was 204 students.

At the time of the survey, Dura Primary School had 562 students, and Debrebrhan Secondary School had 191 students, giving a total enrollment of 753 students. The sample was proportionally allocated to each school according to the number of students; 152 students were selected from Dura Primary School and 52 from Debrebrhan Secondary School. Within each school, students were stratified by grade level (class), and the allocated sample size was further proportionally distributed across classes/grades. A systematic random sampling method was then applied within each class. The sampling interval *k* was calculated as $k=\frac{N}{n_{c}}$, where *N* is the total population of eligible students in each class listed as 1, 2, 3, *N*, and *n*_c_ is the calculated sample size per school. The first student was randomly selected from the class list, and subsequent participants were chosen at regular intervals using the class roster as the sampling frame. Of the 204 students initially selected, 190 students provided sufficient stool samples and were included in the final laboratory analysis.

### 2.6 Data Collection and Processing

Questionnaire survey: Data were collected using a semi‐structured and pretested questionnaire administered by trained data collectors. The questionnaire captured information on: (i) socio‐demographic information (age, gender, educational rank, school); (ii) behavioral and environmental risk factors, including handwashing practices, shoe‐wearing habits, fingernail hygiene, latrine availability and use, and handwashing practice after latrine use. The questionnaire was administered in the local language, Tigrinya, to ensure comprehension.

Parasitological examination: Each participant received a clean, labeled, leak‐proof stool container and provided around 3 g of fresh stool. The samples were then transported in cool boxes to Aksum University Comprehensive Hospital within 4 h of collection. Parasitological examination was conducted using the Kato–Katz technique following standard World Health Organization (WHO) guidelines [33]. Key laboratory procedures included preparing one Kato–Katz smear from each stool sample, use of a 41.7 mg template, examination under light microscopy by trained laboratory technicians within 30–60 min after preparation for hookworm detection to prevent egg clearing; and re‐examination of slides later for detection of other helminth eggs. The presence of parasite eggs was recorded.

### 2.7 Quality Control

Quality control measures included training of data collectors and lab technicians, double–entry verifications of data, and re‐examination of 10% of slides by a senior parasitologist who was blinded to the initial results.

### 2.8 Data Analysis

Data were entered into Epidata and exported to SPSS version 25 for analysis. Descriptive statistics, including frequencies, percentages, means, and standard deviations, were used to summarize the socio‐demographic characteristics of the participants and the prevalence of IPIs. Bivariate and multivariate binary logistic regression models were used to determine the associations of socio‐demographic characteristics and other factors with a 95% confidence level. Initially, all variables were assessed using bivariate analysis. Multicollinearity among independent variables was assessed using correlation coefficients and variance inflation factor (VIF) values prior to multivariable logistic regression analysis. Variables with a *p* value ≤ 0.10 in the bivariate analysis were considered candidates for inclusion in the multivariable logistic regression model. This approach was used to minimize over‐adjustment and to ensure that only relevant variables were included in the final model. The selected variables were then entered into the multivariable logistic regression model to control for potential confounding and to identify independent predictors of infection. Adjusted odds ratios (AORs) with 95% confidence intervals (CIs) were computed to measure the strength of associations. A *p* value of less than 0.05 was considered statistically significant in the final model.

### 2.9 Ethical Approval

Ethical approval for the study was obtained from the Research Ethical Review Committee (RERC) of Hawassa University (reference number: CNCS‐REC019/24), with additional permissions secured from La'elay Maichew District Health and Education authorities and the directors of the selected schools. Participating children were given a detailed explanation of the study's objectives and provided their informed consent before specimen collection. They were informed of their right to withdraw at any time. All collected information was kept confidential. Children diagnosed with IPIs were also referred to nearby health facilities for treatment.

## 3 Results

### 3.1 Socio‐Demographic and Behavioral Characteristics of Participants

A total of 190 students provided complete questionnaire responses and adequate stool samples for parasitological examination, yielding a response rate of 93.1% of the initially selected sample. Slightly more than half of the participants were female (53.2%). The majority of students (65.3%) were aged 7–14 years, and 34.7% were aged 15–21 years. Most students (77.4%) were enrolled in elementary school. Behavioral characteristics showed that 76.3% did not regularly wash their hands both before and after a meal, 75.8% did not use toilet facilities regularly, 82.1% did not wash their hand after toilet use, and 80.5% walking barefoot (Table 1).

**Table 1:** Socio‐demographic and behavioral characteristics of the study participants ([*n* = 190], 2024).

Variable	Category	Total examined *n* (%)	95% CI (%)
Gender	Male	89 (46.8)	39.7–53.9
	Female	101 (53.2)	46.1–60.3
Age (years)	7–14	124 (65.3)	58.5–72.1
	15–21	66 (34.7)	27.9–41.5
Grade level	Grade 1–8	147 (77.4)	71.5–83.3
	Grade 9–12	43 (22.6)	16.7–28.5
School	Dura elementary school	147 (77.4)	71.5–83.3
	Debrebrhan secondary school	43 (22.6)	16.7–28.5
Regular handwashing before/after meals	Yes	45 (23.7)	17.7–29.7
	No	145 (76.3)	70.3–82.3
Barefoot walking	Yes	37 (19.5)	13.9–25.1
	No	153 (80.5)	74.9–86.1
Use of toilet facility	Yes	46 (24.2)	18.1–30.3
	No	144 (75.8)	69.7–81.9
Handwashing habit after toilet use	Yes	34 (17.9)	12.5–23.3
	No	156 (82.1)	76.7–87.5

*Note:* Percentages represent the proportion within each variable category.

### 3.2 Prevalence of Intestinal Parasitic Infections

**Figure 2:**
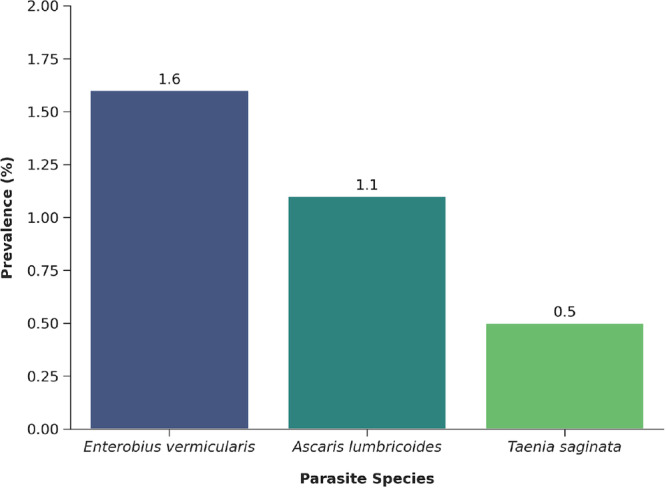
The prevalence of IPIs of school students in Dura *Kebele*, La'elay Maichew district of Tigray region, Ethiopia ([*n* = 190], 2024).

Out of the 190 stool samples examined, six students tested positive for IPIs, resulting in an overall prevalence of 3.2% (95% CI: 1.2–6.9). Three species of IPIs were identified: *Enterobius vermicularis* (1.6%), *Ascaris lumbricoides* (1.1%), and *Taenia saginata* (0.5%) (Figure 2).

### 3.3 Distribution of Infection by Socio‐Demographic and Behavioral Characteristics

Infection prevalence was slightly higher among males (3.4%) than among females (3%). Age‐specific analysis showed that students aged 15–21 years had a higher prevalence rate (7.6%) compared with those aged 7–14 years (0.8%). Similarly, infection prevalence was higher among secondary school students (9.3%) compared with elementary school students (1.4%). With respect to behavioral factors, infections were observed in both groups regardless of hygiene practices. For example, infection prevalence was slightly higher among students who did not regularly use toilet facilities, compared with 2.2% among those who reported toilet use (Table 2).

**Table 2:** Prevalence of IPIs across the different socio‐demographic and behavioral characteristics of the study participants ([*n* = 190], 2024).

Variables	Category	Examined *n* (%)	Positive *n* (%)	95% CI (%)
Age (years)	7–14	124 (65.3)	1 (0.8)	0.0–2.4
	15–21	66 (34.7)	5 (7.6)	1.2–14.0
Gender	Male	89 (46.8)	3 (3.4)	0.0–7.1
	Female	101 (53.2)	3 (3)	0.0–6.3
Grade level	Grade 1–8	147 (77.3)	2 (1.4)	0.0–3.2
	Grade 9–12	43 (22.3)	4 (9.3)	0.6–18.0
Regular handwashing before/after meals	Yes	45 (23.7)	2 (4.4)	0.0–10.4
	No	145 (76.3)	4 (2.8)	0.1–5.4
Barefoot walking	Yes	37 (19.5)	1 (2.7)	0.0–7.9
	No	153 (80.5)	5 (3.3)	0.5–6.1
Use of toilet facility	Yes	46 (24.2)	1 (2.2)	0.0–6.4
	No	144 (75.8)	5 (3.5)	0.5–6.5
Handwashing habit after toilet use	Yes	34 (17.9)	1 (2.9)	0.0–8.6
	No	156 (82.1)	5 (3.2)	0.4–6.0

*Note:* Percentages represent the proportion within each variable category.

### 3.4 Potential Predictors of Soil‐Transmitted Helminth Infection

Table 3 displays the results of a logistic regression model investigation of potential risk factors for IPIs. In the univariate analysis, school students aged 7–14 had a lower odds of IPIs compared to those aged 15–21 years (COR 0.09; 95% CI: 0.01–0.87). Similarly, children in grades 1–8 had lower odds of IPIs compared to those in grades 9–12 (COR = 0.13, 95% CI: 0.02–0.76). However, in multivariate logistic regression, the adjusted odds ratios (AOR) changed substantially (e.g., age 7–14: AOR = 5.25; 95% CI: 0.29–96.46; *p* = 0.264), reflecting the very low number of positive cases (only six events). This sparse‐data bias makes the multivariate analysis unreliable and underpowered to detect true associations. While no statistically significant associations were observed in the adjusted model, these results should be interpreted with caution.

**Table 3:** Predicted risk factors of IPIs among the school students attending primary and secondary schools in Dura Keble, La'elay Maichew woreda, Northern Ethiopia ([*n* = 190], 2024).

Variables	Category	IPIs status		COR (95% CI)	*p* value	AOR (95% CI)	*p* value
		Negative	Positive				
Gender	Male	86 (96.6)	3 (3.4)	1.14 (0.22–5.79)	0.875	—	—
	Female	98 (97)	3 (3)	Ref		—	
Age (years)	7–14	123 (99.2)	1 (0.8)	**0.09 (0.01–0.87)**	0.037	0.18 (0.01–2.97)	0.230
	15–21	61 (92.4)	5 (7.6)	Ref		Ref	
Grade level	Grade 1–8	145 (98.6)	2 (1.4)	**0.13 (0.02–0.76)**	0.023	0.44 (0.05–4.22)	0.479
	Grade 9–12	39 (90.7)	4 (9.3)	Ref		Ref	
Regularly handwashing before/after meals	Yes	43 (95.6)	2 (4.4)	0.92 (0.11–8.09)	0.936	—	—
	No	141 (97.2)	4 (2.8)	Ref		—	
Barefoot walking	Yes	36 (97.3)	1 (2.7)	0.82 (0.09–7.26)	0.860	—	—
	No	148 (96.7)	5 (3.3)	Ref		—	
Use toilet facility	Yes	45 (97.8)	1 (2.2)	0.62 (0.07–5.43)	0.664	—	—
	No	139 (96.5)	5 (3.5)	Ref		—	
Hand washing habit after toilet use	Yes	33 (97.1)	1 (2.9)	1.64 (0.29–9.26)	0.576	—	—
	No	151 (96.8)	5 (3.2)	Ref		—	

*Note:* Bold values indicate statistical significance (*p *< 0.05).

Abbreviations: AOR, adjusted odds ratio; CI, confidence interval; COR, crude odds ratio; IPIs, intestinal parasitic infections; Ref, reference category. (—: The variables not included in multivariable models).

## 4 Discussion

The study assessed the prevalence and associated risk factors of IPIs among students attending two schools in Dura *Kebele*, La'elay Maichew district, northern Ethiopia. Among the 190 students examined, six were infected with IPIs, yielding an overall prevalence of 3.2%. Three parasite species were identified: *E. vermicularis* (1.6%), *A. lumbricoides* (1.1%), and *T. saginata* (0.5%). This finding may reflect the impact of previous MDA campaigns and WASH interventions in the study area.

From an epidemiological perspective, the prevalence observed in this study is substantially lower than national and regional estimates reported in Ethiopia over the past decade. A recent systematic review and meta‐analysis reported pooled prevalence estimates of IPIs among Ethiopian children ranging between approximately 39% and 54%, indicating that IPIs remain a significant public health problem in many parts of the country [34]. Similarly, studies conducted in different Ethiopian regions have documented markedly higher prevalence levels among school children, including Ambesame (31.2%) [35], Birbir town (27.1%) [36], Mekaneselam (12.6%) [37], Butajira (25%) [2], Wukro (60.7%) [20], Arbaminch Zuria [38, 39], and Jawi town [40]. Similar higher prevalence levels have been documented in other Ethiopian districts such as Doreni [41] and Kola Diba [42]. Despite similar rural settings, the lower prevalence observed in this study may reflect differences in intervention coverage and timing, variations in study period, diagnostic methods, environmental conditions, and population characteristics.

Conversely, the prevalence observed in this study is similar to findings reported in nearby areas, including Medebay Zana district in Tigray [19], where low infection levels have also been documented. Several factors may explain the relatively low prevalence observed in the present study. Ethiopia has implemented nationwide preventive chemotherapy programs targeting STHs among SAC since 2013 [8]. These MDA campaigns, together with ongoing health education and improvements in sanitation, may have contributed to reducing infection levels in many areas. However, the low prevalence observed in this study should not be attributed solely to the success of prior MDA programs, particularly given that routine deworming activities were interrupted for nearly 3 years during the recent conflict in Tigray. Other explanations should also be considered. For instance, the use of a single stool sample, the limited sensitivity of the Kato–Katz technique for detecting light infections, and the relatively small sample size may have contributed to an underestimation of the true prevalence. The low prevalence may also reflect post‐conflict population displacement and changes in school attendance patterns. In addition, the study included students from only two schools within one *kebele*, which may limit the representativeness of the wider district population. Similarly low prevalence rates have also been documented in various international settings [43, 44, 45, 46, 47, 48, 49, 50, 51, 52, 53]. Nevertheless, the reported prevalence remains considerably lower than those documented in several other countries, such as Ghana [54], Uganda [55], and Afghanistan [56], highlighting geographic disparities that may be influenced by differences in environmental conditions, health infrastructure, and the implementation of control programs.

Another notable finding was the predominance of *E. vermicularis* among the detected parasite species. Unlike STHs, *E. vermicularis* is primarily transmitted through direct person‐to‐person contact and contaminated surfaces, particularly in crowded settings such as schools [57]. The mode of transmission highlights the importance of improving personal hygiene and environmental sanitation in schools, including handwashing, nail hygiene, and regular cleaning of shared surfaces.

### 4.1 Limitations of the Study

Several limitations should be considered when interpreting these findings. First, the study relied on a single stool sample per participant and used only the Kato–Katz thick smear technique for parasitological examination. While recommended for detecting STHs, Kato–Katz has limited sensitivity for low‐intensity infections and certain protozoan parasites, and examining multiple samples or using complementary methods (e.g., formalin‐ether concentration) could have improved diagnostic accuracy. Additionally, the adhesive tape test, the preferred method for detecting *E. vermicularis*, was not employed, likely resulting in underestimation of its prevalence. Second, the small number of positive cases limited the statistical power of regression analyses, making the multivariable models underpowered and potentially unreliable for detecting associations between behavioral risk factors and infection status. Third, the study did not assess infection intensity (eggs per gram of stool, EPG), which is critical for evaluating the burden of helminth infections and the potential impact of mass drug administration programs. Fourth, the study was conducted in only two schools within a single *kebele*, which may not be representative of the broader La'elay Maichew district or the wider Tigray region. Fifth, post‐conflict disruptions in school attendance may have introduced selection bias; children who remained in school may have been healthier or come from more stable households, which could affect the observed prevalence and risk patterns. Finally, the study assessed only a limited number of behavioral and environmental factors, and future research should include larger sample sizes, multiple schools, repeated stool sampling, more sensitive diagnostic techniques, measures of infection intensity, and broader environmental and socio‐demographic variables to provide a more comprehensive understanding of parasite transmission dynamics in the region.

## 5 Conclusions

The study revealed a low prevalence (3.2%) of intestinal parasitic infections among students in Dura *Kebele*, La'elay Maichew District, with *E. vermicularis*, *A. lumbricoides*, and *T. saginata* detected among the infected participants, which may reflect the impact of prior MDA campaigns and WASH initiatives in the study area. Although no statistical associations were identified, the persistence of poor hygiene practices and the detection of *E. vermicularis* (which spreads directly) suggest that integrated WASH and hygiene education remain important.

## Author Contributions

**Teklay Abrha Yanshet:** conceptualization, investigation, funding acquisition, writing – original draft, methodology, validation, visualization, writing – review and editing, software, formal analysis, project administration, data curation, supervision, and resources. **Gebremedhin Gebrezgabiher:** investigation, conceptualization, writing – original draft, methodology, validation, visualization, writing – review and editing, software, formal analysis, data curation, and supervision. **Melese Birmeka:** conceptualization, investigation, funding acquisition, writing – original draft, methodology, validation, visualization, writing – review and editing, software, formal analysis, project administration, data curation, supervision, and resources.

## Conflicts of Interest

The authors declare no conflicts of interest.

## Transparency Statement

The corresponding author, Gebremedhin Gebrezgbaiher, affirms that this manuscript is an honest, accurate, and transparent account of the study being reported; that no important aspects of the study have been omitted; and that any discrepancies from the study as planned have been explained.

## Data Availability

The data sets used for this study are available from the corresponding author upon reasonable request.
